# Toward a Tripartite Taxonomy of Entropy in Physics

**DOI:** 10.3390/e28060704

**Published:** 2026-06-18

**Authors:** Antoine Druilhe

**Affiliations:** Électricité de France (EDF) R&D, 78401 Chatou, France; antoine.druilhe@edf.fr

**Keywords:** entropy, second law, quantum information, black hole thermodynamics, Bekenstein bound, emergent thermodynamics

## Abstract

The term “entropy” denotes several mathematically distinct quantities across modern physics, including thermodynamic, statistical, quantum-informational, and geometric notions that are often conflated in foundational discussions. We propose an operational distinction among three such quantities: a geometric capacity entropy $S_{\mathrm{capacity}}$ proportional to a region’s bounding area, a microscopic content entropy $S_{\mathrm{content}}$ given by the fine-grained von Neumann entropy of the reduced state, and a thermodynamic entropy $S_{\mathrm{thermo}}$ corresponding to the observer-relative ignorance that remains after accessible information is accounted for. We argue that keeping these quantities distinct is not merely terminological: within this framework, the second law of thermodynamics can be formulated as a typical consequence of unitary dynamics combined with bounded observational access, rather than as an independent postulate. The distinction also clarifies which entropy enters established results such as the Bekenstein–Hawking entropy of black holes and the Clausius relation in Jacobson’s thermodynamic derivation of Einstein’s equations. The proposed framework is conceptual and does not modify established physical theories; it is intended as a useful clarification for informational approaches to physics.

## 1. Introduction

The word entropy has been used in physics for more than a century, and it has acquired several meanings along the way. Boltzmann gave it a statistical formulation $S_{B}=k_{B}\ln W$, with *W* the number of microstates compatible with the macroscopic constraints [1]. Clausius defined a thermodynamic entropy through $dS=\delta Q_{\mathrm{rev}}/T$ along reversible paths [2]. Shannon introduced an information-theoretic measure $H=-\sum_{i}p_{i}\log p_{i}$ for probability distributions [3]. Von Neumann gave the quantum generalization $S_{vN}=-\mathrm{Tr}\left(\rho \ln\rho \right)$ [4], and Bekenstein the geometric area entropy [5]. These quantities share a name but are mathematically distinct. Practitioners usually navigate the multiplicity without difficulty within their own domain. Confusion arises when results from different domains meet—most visibly in black hole thermodynamics, in the foundations of statistical mechanics, and in informational approaches to gravity.

A representative example is the relation

(1) $$S_{BH}\;=\;\frac{A}{4\ell_{P}^{2}},$$

where $S_{BH}$ is the Bekenstein–Hawking entropy of a black hole, *A* is the area of its event horizon, and $\ell_{P}=\sqrt{G\hbar /c^{3}}$ is the Planck length [5,6]. Throughout the paper we use units in which $k_{B}=1$; restoring conventional units yields $S_{BH}=k_{B}A/\left(4\ell_{P}^{2}\right)$, with *S* measured in nats unless otherwise stated. Equation (1) is universally called an entropy. What is less clear is whether it should be read as the von Neumann entropy of an underlying microstate, as a thermodynamic entropy in the sense of Clausius, or as a geometric upper bound on the information content of the region. The reading chosen has physical consequences, and feeds into long-standing debates about the black hole information paradox [7,8].

A similar situation arises in Jacobson’s derivation of Einstein’s equations from the Clausius relation [9]. There, the relation

(2) δQ=TdS

is applied to local Rindler horizons, with *S* identified with the area entropy and *T* with the Unruh temperature. We follow the standard notation: δQ denotes an inexact differential (path-dependent heat exchange), dS an exact differential (state function increment), and *T* acts as the integrating factor that makes δQ/T exact along quasi-static reversible processes. The derivation is technically clean and conceptually suggestive. But which entropy is *S*? It is the area entropy (1)—yet the Clausius relation itself is a thermodynamic statement. Whether the area entropy is thermodynamic in the relevant sense is the conceptual issue that needs to be clarified.

The aim of the present paper is to propose an operational distinction among three entropy-like quantities that are commonly conflated, and to examine the consequences of keeping them distinct. The proposed taxonomy consists of

1. A capacity entropy $S_{\mathrm{capacity}}$, set by the geometric bound of Bekenstein–Hawking type, representing the maximum information content compatible with the geometry of a region.
2. A content entropy $S_{\mathrm{content}}$, given by the fine-grained von Neumann entropy of the reduced quantum state of the region, representing the actual quantum-informational content of the state.
3. A thermodynamic entropy $S_{\mathrm{thermo}}$, defined relative to an observer with finite measurement resolution, as the content entropy minus the information accessible through that observer’s measurements.

The principal claim of this paper is that this distinction is more than terminological. Once the three quantities are kept separate, several conceptual issues become tractable. In particular, we show that the second law of thermodynamics, formulated as a statement about $S_{\mathrm{thermo}}$, emerges as a typical consequence of unitary quantum dynamics combined with the boundedness of observational access. It does not require a separate axiomatic postulate.

The introduction of the observer in the definition of $S_{\mathrm{thermo}}$ is not an anthropic move. It situates the framework within the information-theoretic tradition of statistical mechanics initiated by Jaynes [10], formalized through Landauer’s principle relating information erasure to thermodynamic dissipation [11], articulated by Bennett in the analysis of Maxwell’s demon [12], and developed in the modern field of quantum thermodynamics [13,14]. In all these frameworks, the thermodynamic entropy is operationally tied to the information available to a specified observer or measurement apparatus and kept distinct from the fine-grained microscopic entropy of the underlying state. What the taxonomy proposed here does is to make that distinction explicit at the level of definitions, instead of leaving it implicit.

A few words on what this paper does not claim. The taxonomy proposed here is conceptual. It is not a new dynamical theory. It does not modify any established result in quantum mechanics, statistical mechanics, or general relativity. It predicts no new physical phenomena. The intended contribution is a clarification that may help in foundational discussions where the three entropies have been conflated.

The rest of the paper is organized as follows. Section 2 gives the formal definitions of the three entropies and the structural inequalities relating them, together with a concrete operational example. Section 3 establishes the emergent character of the second law in this framework, with the operational regime specified explicitly. Section 4 applies the taxonomy to two contexts where the distinction matters interpretively: black hole thermodynamics and Jacobson’s derivation. Section 5 places the framework within the existing literature on information-theoretic and quantum gravitational entropy. Section 6 discusses limitations and possible extensions, and Section 7 concludes.

## 2. The Three Entropies

We work throughout in a fixed quantum-mechanical setting. The universe is described by a global Hilbert space H in some pure state |Ψ〉, evolving unitarily under a Hamiltonian *H*. A region *R* is a bounded subset of physical space. To it we associate a sub-Hilbert space $H_{R}$ through the standard tensor-product decomposition $H=H_{R}⊗H_{\bar{R}}$. The reduced state on *R* is

(3) $$\rho_{R}\;=\;{\mathrm{Tr}}_{\bar{R}}\;|\Psi \rangle \langle \Psi |,$$

where the partial trace ${\mathrm{Tr}}_{\bar{R}}$ is taken over the complementary Hilbert space $H_{\bar{R}}$ associated with the complement region $\bar{R}$. The reduced density matrix $\rho_{R}$ describes the statistical properties of measurements performed entirely within *R*, while ignoring outcomes in $\bar{R}$.

We assume that this decomposition is well-defined. In gauge theories and gravity it is not generally so: local subsystems carry edge modes, and the algebra of observables associated with a region is typically not of type I in the von Neumann algebraic classification [15,16]. The proper framework in those cases involves type II or type III algebras, with their own subtleties in defining entropy. Here we restrict attention to regimes where the tensor-product decomposition is operationally meaningful: finite-dimensional approximations, regulated quantum field theories with type I factorization, lattice models. This restriction matters for fully covariant extensions of the framework, which we return to in Section 6.

### 2.1. Capacity Entropy $S_{\mathrm{capacity}}$

The capacity entropy of a region *R* is defined as the Bekenstein–Hawking quantity

(4) $$S_{\mathrm{capacity}}\left(R\right)\;\equiv \;\frac{A(R)}{4\ell_{P}^{2}},$$

where A(R) is the area of the boundary ∂R. We read $S_{\mathrm{capacity}}$ as a geometric upper bound on the information that can be encoded in the region.

Several related concepts in the literature deserve to be distinguished, since they are sometimes conflated.

The Bekenstein–Hawking entropy is the specific quantity $S_{BH}=A_{\mathrm{horizon}}/\left(4\ell_{P}^{2}\right)$ attached to a black hole horizon, derived from the laws of black hole mechanics combined with Hawking radiation [5,6,17]. It is a particular case of the general capacity entropy (4) when *R* is the region enclosed by an event horizon.

The Bekenstein bound is the inequality $S\leq 2\pi k_{B}ER/\left(\hbar c\right)$ relating the entropy of a bounded system of energy *E* and radius *R* to its energy and size [18]. It is a bound on actual entropy, energy-dependent, and does not by itself assert proportionality to area.

The holographic bound (sometimes called the spherical entropy bound), formulated by ’t Hooft [19] and Susskind [20], is the statement that the total entropy of any region is bounded by the area of its boundary in Planck units, $S\leq A/(4\ell_{P}^{2})$.

The Bousso covariant entropy bound is a refinement of the holographic bound that uses light-sheets rather than spacelike surfaces and is conjectured to apply in arbitrary curved spacetimes under specified geometric conditions [21,22].

In the present taxonomy, $S_{\mathrm{capacity}}$ as defined in (4) is meant to capture the conceptual content shared by the holographic bound and the Bekenstein–Hawking entropy: a geometric upper bound proportional to the boundary area. We do not claim that (4) holds in full generality for arbitrary bounded regions in curved spacetime. In such cases, the more refined covariant Bousso bound is the proper formulation. The simpler form (4) suffices for our conceptual purposes, with the understanding that the extension to covariant settings is governed by the established literature on covariant entropy bounds.

A point of terminology is in order. The quantity $S_{\mathrm{capacity}}$ is often called simply “the entropy of the region” in the literature. We find this usage misleading: it suggests a direct correspondence between geometric area and a thermodynamic or microscopic entropy, which is a substantive claim and requires justification beyond the bound itself. Calling the quantity a capacity entropy stresses that it is an upper bound on possible content, not a statement about actual content.

### 2.2. Content Entropy $S_{\mathrm{content}}$

The content entropy of a region *R* is the fine-grained von Neumann entropy of the reduced state:

(5) $$S_{\mathrm{content}}\left(R\right)\;\equiv \;-\mathrm{Tr}\;\left(\rho_{R}\ln\rho_{R}\right).$$

We read $S_{\mathrm{content}}$ as the fine-grained subsystem entropy of the region’s reduced state. When the global state is pure, $S_{\mathrm{content}}\left(R\right)$ quantifies the entanglement between *R* and its complement $\bar{R}$. We avoid the more colloquial phrase “the information content of the region”—which would suggest a quantity defined intrinsically by what is encoded inside *R*—because for a pure global state, $S_{\mathrm{content}}\left(R\right)$ depends on correlations with the exterior and is not localized within *R* in any elementary sense.

When the reduced state $\rho_{R}$ is well-approximated by a Gibbs state $\propto e^{-\beta H_{R}}$ with effective inverse temperature β and effective local Hamiltonian $H_{R}$, $S_{\mathrm{content}}\left(R\right)$ coincides with the standard thermodynamic entropy of the equilibrium ensemble. The two notions agree in the thermal regime; they differ in general, whenever the reduced state retains coherent quantum correlations with the exterior beyond a thermal description.

The content entropy is the natural object in quantum information theory [23], in the generalized second law [24], and in the holographic computation of entanglement entropy via the Ryu–Takayanagi prescription and its quantum corrections [25,26,27]. It is operationally meaningful: given unlimited resources and arbitrary measurement capabilities, an observer with access to all of *R* could reconstruct $\rho_{R}$ and compute $S_{\mathrm{content}}$ tomographically.

### 2.3. Relation to Boltzmann and Shannon Entropies

For clarity, we situate the proposed taxonomy with respect to the classical entropy concepts mentioned in the introduction.

The Boltzmann entropy $S_{B}=k_{B}\ln W$ counts the number of microstates compatible with a coarse-grained macroscopic state. Quantum-statistically, $S_{B}$ corresponds to the coarse-grained entropy of an ensemble determined by macroscopic constraints. It is naturally aligned with $S_{\mathrm{content}}$ when the relevant ensemble is approximately thermal: the von Neumann entropy of the thermal state reduces to the Boltzmann entropy in the appropriate limit. The Boltzmann entropy, however, is defined relative to a specified coarse-graining (typically energy macrostates), which introduces an implicit observer-like element through the choice of macroscopic variables. In that sense, $S_{B}$ sits operationally between $S_{\mathrm{content}}$ (no coarse-graining beyond the partial trace defining *R*) and $S_{\mathrm{thermo}}$ (explicit observer-relativity through accessible measurements).

The Shannon entropy $H=-\sum_{i}p_{i}\log p_{i}$ is the information-theoretic measure attached to a probability distribution. The von Neumann entropy in (5) reduces to the Shannon entropy of the eigenvalue distribution of $\rho_{R}$. In this precise sense, $S_{\mathrm{content}}$ is the natural quantum generalization of the Shannon entropy applied to the reduced state. Conversely, $I_{\mathrm{accessible}}$, defined below, is a Shannon-type quantity associated with the classical probability distribution generated by the observer’s measurement protocol.

The taxonomy proposed here does not replace these classical entropies; it organizes their quantum-mechanical extensions according to the operational role each plays in the description of a region of space.

### 2.4. The Structural Inequality

A consistent quantum theory of gravity is generally expected to respect the bound

(6) $$S_{\mathrm{content}}\left(R\right)\;\leq \;S_{\mathrm{capacity}}\left(R\right).$$

This is the operational form of the holographic bound [19,20], here recast as a relation between two distinct entropy-like quantities rather than as a single statement about a single entropy.

Two clarifications are needed for the inequality to be operationally precise. First, the inequality is to be read in the regulated or coarse-grained sense: the strict von Neumann entropy of a continuum quantum field theory on a bounded region typically diverges, owing to short-distance entanglement across the boundary. A regularization—lattice cutoff, smearing of the boundary, vacuum subtraction—is required to obtain finite, comparable quantities. Second, saturation of (6) is often conjectured at black hole horizons in semiclassical treatments, where the relevant comparison is between regulated entanglement entropy and the horizon area in Planck units.

We do not derive (6) here. It is adopted as a structural input grounded in the established literature. Its operational role is to express that $S_{\mathrm{capacity}}$ bounds $S_{\mathrm{content}}$ without, in general, equaling it. The two are conceptually independent quantities tied by a fundamental relation.

### 2.5. Thermodynamic Entropy $S_{\mathrm{thermo}}$

The thermodynamic entropy is defined relative to an observer O with specified measurement capabilities. Let $I_{\mathrm{accessible}}\left(R,O\right)$ denote the information about the state of *R* that O can operationally access through the measurements at its disposal. We define

(7) $$S_{\mathrm{thermo}}\left(R,O\right)\;\equiv \;S_{\mathrm{content}}\left(R\right)\;-\;I_{\mathrm{accessible}}\left(R,O\right).$$

We define $I_{\mathrm{accessible}}$ operationally as the mutual information that O can in principle extract through measurements on the accessible degrees of freedom. Its precise value depends on the observer’s measurement capabilities and on the operational protocol used. For our purposes here, only the structural inequality

(8) $$I_{\mathrm{accessible}}\left(R,O\right)\;\leq \;I{\left(R:O\right)}_{\rho }$$

is required, where $I{\left(R:O\right)}_{\rho }=S\left(\rho_{R}\right)+S\left(\rho_{O}\right)-S\left(\rho_{RO}\right)$ is the quantum mutual information between the region and the observer in the global state ρ. All quantities in (7) and (8) are measured in the same units (nats with our convention $k_{B}=1$).

### 2.6. Operational Example: A Two-Spin Model

To make $I_{\mathrm{accessible}}$ concrete, we work through the simplest non-trivial example.

Consider a system *R* consisting of a single qubit, entangled with an environment $\bar{R}$ also reduced to a single qubit, in the global pure state

(9) $$|\Psi \rangle \;=\;\cos\theta |0_{R}0_{\bar{R}}\rangle +\sin\theta |1_{R}1_{\bar{R}}\rangle ,\;\theta \in [0,\pi /4].$$

The reduced state on *R* is diagonal in the {|0〉,|1〉} basis with eigenvalues $p_{0}=\cos^{2}\theta$ and $p_{1}=\sin^{2}\theta$. The content entropy is

(10) $$S_{\mathrm{content}}\left(R\right)\;=\;-p_{0}\ln p_{0}-p_{1}\ln p_{1}\;=\;h\left(\cos^{2}\theta \right),$$

with h(x)=−xlnx−(1−x)ln(1−x) the binary entropy function. For θ=π/4 (maximal entanglement), $S_{\mathrm{content}}\left(R\right)=\ln2$.

Now, consider an observer O who can perform a single projective measurement of the observable ${\hat{O}}_{z}=\sigma_{z}$ on *R*, with outcomes ±1 corresponding to |0〉,|1〉. The probability distribution of outcomes is $\left(p_{0},p_{1}\right)$, and the Shannon entropy of this distribution equals $h(p_{0})$. The mutual information that O extracts about the state through this measurement equals $h(p_{0})$, since the measurement is informationally complete with respect to the diagonal density matrix:

(11) $$I_{\mathrm{accessible}}\left(R,O_{\sigma_{z}}\right)\;=\;h\left(p_{0}\right)\;=\;S_{\mathrm{content}}\left(R\right),$$

and therefore $S_{\mathrm{thermo}}\left(R,O_{\sigma_{z}}\right)=0$. The observer adapted to the local basis sees no residual ignorance.

Now, consider a different observer $O^{\prime }$ who can only measure ${\hat{O}}_{x}=\sigma_{x}$. Each outcome has equal probability 1/2 regardless of θ. The measurement is independent of θ for any θ and provides no information distinguishing the eigenstructure of $\rho_{R}$:

(12) $$I_{\mathrm{accessible}}\left(R,O_{\sigma_{x}}^{\prime }\right)\;=\;0,$$

hence $S_{\mathrm{thermo}}\left(R,O_{\sigma_{x}}^{\prime }\right)=S_{\mathrm{content}}\left(R\right)=h\left(\cos^{2}\theta \right)$.

The two observers measure the same physical system and assign different thermodynamic entropies: zero for one, the full content entropy for the other. The example shows the operational content of (7): $S_{\mathrm{thermo}}$ depends on what the observer can actually distinguish. The structural inequality $I_{\mathrm{accessible}}\leq I{\left(R:O\right)}_{\rho }$ is saturated when the observer’s measurement basis is aligned with the eigenbasis of $\rho_{R}$ and the observer has dimensional access matching $\rho_{R}$; it is strictly violated upward only by suboptimal measurement choices.

The interpretation of $S_{\mathrm{thermo}}$ is the standard thermodynamic one: it is the ignorance of the observer about the microstate, given the macroscopic information at the observer’s disposal. The new content of (7) is its explicit relative character. The thermodynamic entropy is not an intrinsic property of the region; it is a property of the region–observer pair. Different observers, with different measurement capabilities, assign different values of $S_{\mathrm{thermo}}$ to the same region. This is consistent with Jaynes’s view of thermodynamic entropy as an information-theoretic quantity [10], with Landauer’s identification of information erasure as the thermodynamic cost of measurement [11], and with modern treatments of quantum thermodynamics [13,14]. What the present taxonomy adds is to make the separation from $S_{\mathrm{content}}$ a defining relation, rather than an implicit consequence.

### 2.7. Summary

The three entropies and their relations are summarized in Table 1.

The structural relations among them are:

(13) $$\begin{array}{cccc} S_{\mathrm{content}}\left(R\right)\; & \leq \;S_{\mathrm{capacity}}\left(R\right) & \; & (\mathrm{holographic}\;\mathrm{bound}), \end{array}$$

(14) $$\begin{array}{cccc} S_{\mathrm{thermo}}\left(R,O\right)\; & =\;S_{\mathrm{content}}\left(R\right)-I_{\mathrm{accessible}}\left(R,O\right) & \; & \left(\mathrm{observer}-\mathrm{relative}\right), \end{array}$$

(15) $$\begin{array}{cccc} I_{\mathrm{accessible}}\left(R,O\right)\; & \leq \;I{\left(R:O\right)}_{\rho } & \; & (\mathrm{operational}\;\mathrm{bound}). \end{array}$$

## 3. The Second Law as an Emergent Typical Statement

The second law of thermodynamics asserts that the entropy of a closed system tends to increase. In the framework of the previous section, the relevant quantity is $S_{\mathrm{thermo}}$, since the second law is an empirical statement about thermodynamic behavior as observed by finite-resolution observers. The argument of this section is that the second law, so formulated, emerges as a typical consequence of unitary quantum dynamics combined with bounded observational access, rather than requiring an independent postulate.

### 3.1. Operational Regime of the Derivation

To avoid ambiguity, we specify the operational regime in which the derivation applies. The argument assumes:

1. A finite-dimensional Hilbert space $H=H_{R}⊗H_{\bar{R}}$ with $\dim H_{R}\ll \dim H_{\bar{R}}$. The system *R* is small relative to its environment.
2. A Hamiltonian *H* drawn from the Gaussian Unitary Ensemble (GUE), or more generally a non-integrable Hamiltonian satisfying the eigenstate thermalization hypothesis [28,29].
3. An initial state $|\Psi (0)\rangle =|\psi_{R}\left(0\right)\rangle ⊗|\psi_{\bar{R}}\left(0\right)\rangle$ that is factorized between *R* and $\bar{R}$.
4. A time window $\left[0,t_{\mathrm{eq}}\right]$, where $t_{\mathrm{eq}}$ is the equilibration time of the system characteristic of the Hamiltonian and the initial state, with $t_{\mathrm{eq}}$ much smaller than the Heisenberg recurrence time $t_{H}∼e^{S}$ of the global system.
5. A fixed observer O with a measurement apparatus characterized by a maximum mutual information $I_{\max}\left(O\right)$ that bounds the information extractable from *R* regardless of measurement protocol.

These are the standard assumptions under which the typicality and equilibration results of quantum statistical mechanics apply [30,31,32]. The argument does not aim to derive the second law for arbitrary dynamics. It shows only that within these well-defined assumptions, the second law follows from quantum-mechanical and informational ingredients, with no additional postulate.

### 3.2. Behavior of $S_{\mathrm{content}}$ Under Unitary Evolution

For a closed system in a pure state |Ψ〉 evolving under a unitary $U=e^{-iHt/\hbar }$, the global state remains pure and its von Neumann entropy stays identically zero. The content entropy of a subregion *R*, given by $S_{\mathrm{content}}\left(R\right)=-\mathrm{Tr}\left(\rho_{R}\ln\rho_{R}\right)$, is generally a nontrivial function of time, and not monotonic in general.

Under the assumptions stated above, the typical-evolution result of Popescu, Short, Winter [30] and the canonical typicality result of Goldstein et al. [31] together imply that the subsystem entropy converges, in expectation, toward the equilibrium value

(16) $$S_{\mathrm{content}}^{\mathrm{eq}}\left(R\right)\;=\;\ln d_{R}\;-\;O\left(d_{R}/d_{\bar{R}}\right),$$

with $d_{R}=\dim H_{R}$ and $d_{\bar{R}}=\dim H_{\bar{R}}$. For the regime of interest where $d_{R}\ll d_{\bar{R}}$, this equilibrium value approaches $\ln d_{R}$, which is the maximum entropy compatible with the dimension of *R*. The result is

(17) $$E\;\left[S_{\mathrm{content}}\left(R,t\right)\right]\;\geq \;E\;\left[S_{\mathrm{content}}\left(R,0\right)\right]\;\mathrm{for}\;t\in \left[t_{\mathrm{eq}},t_{H}\right],$$

where the expectation is over typical Hamiltonians in the specified class, or equivalently over typical initial environment states. In the time window $\left[0,t_{\mathrm{eq}}\right]$, the entropy grows from its initial value toward (16); in the window $\left[t_{\mathrm{eq}},t_{H}\right]$, it remains near equilibrium with small fluctuations; for $t≳t_{H}$, recurrence effects become significant and the regime of validity terminates.

In black hole contexts, the analogous timescale beyond which the typicality argument requires modification is the Page time [33], after which the entanglement entropy approaches its maximum and information-theoretic considerations need finer treatment. For the standard thermodynamic regime considered here (*R* much smaller than $\bar{R}$, observation times much shorter than recurrence times), the growth phase (17) is the relevant effective description.

### 3.3. Bounded Growth of $I_{\mathrm{accessible}}$

The information $I_{\mathrm{accessible}}\left(R,O\right)$ accessible to a finite-resolution observer is bounded by the observer’s measurement capabilities. For a fixed observer with fixed measurement apparatus, $I_{\mathrm{accessible}}$ is upper-bounded by a constant $I_{\max}$ reflecting the apparatus’ resolution and total sensitivity:

(18) $$I_{\mathrm{accessible}}\left(R,O,t\right)\;\leq \;I_{\max}\left(O\right).$$

This bound holds at all times, regardless of how the system evolves. The observer may acquire more information over time through repeated measurements, but the total accessible information stays limited by the finite capabilities of the apparatus. The two-spin example of Section 2.6 makes the point concretely: even an optimal observer with measurement basis aligned to $\rho_{R}$ extracts at most $S_{\mathrm{content}}\left(R\right)$; a suboptimal observer extracts strictly less. For a fixed apparatus, the upper bound is structural.

### 3.4. The Emergent Second Law

Taking the time derivative of (7) gives

(19) $$\frac{dS_{\mathrm{thermo}}}{dt}\;=\;\frac{dS_{\mathrm{content}}}{dt}\;-\;\frac{dI_{\mathrm{accessible}}}{dt}.$$

In the time window $\left[0,t_{\mathrm{eq}}\right]$, $dS_{\mathrm{content}}/dt>0$ in expectation, by the typicality result (17). In the same window, $dI_{\mathrm{accessible}}/dt$ is bounded above by the rate at which the apparatus can extract information. By the bound (18), this rate must asymptote to zero on timescales longer than the apparatus saturation time. For sufficiently long times, or whenever the increase in $S_{\mathrm{content}}$ dominates the variation in $I_{\mathrm{accessible}}$, the thermodynamic entropy typically increases:

(20) $$E\;\left[\frac{dS_{\mathrm{thermo}}\left(R,t,O\right)}{dt}\right]\;\geq \;0\;\mathrm{for}\;\mathrm{typical}\;\mathrm{evolution}\;\mathrm{and}\;\mathrm{fixed}\;\mathrm{observer},\;t\in \left[0,t_{\mathrm{eq}}\right].$$

This is the second law of thermodynamics in the framework of the present paper. Three observations are in order.

The second law is not an independent postulate within the stated regime. The result (20) follows from two ingredients: the typical growth of entanglement entropy under unitary dynamics (17), and the boundedness of observational access (18). Both are grounded in established results in quantum information theory, together with operational constraints on finite observers. Within the regime stated in Section 3.1, no additional thermodynamic postulate is needed. We do not claim that the second law in its broadest empirical form—arbitrarily complex thermodynamic transformations, macroscopic systems with collective behavior—is universally reducible to these two ingredients. The claim is only that within the operational regime specified, the reduction is complete.

The second law is observer-relative. The thermodynamic entropy $S_{\mathrm{thermo}}$ depends on the observer through $I_{\mathrm{accessible}}$. Different observers, with different measurement capabilities, assign different values of $S_{\mathrm{thermo}}$ to the same physical system. The two-spin example of Section 2.6 gives the most direct illustration: the same physical state and dynamics yield different thermodynamic entropies for observers measuring different bases. In the limit of an idealized observer with unbounded measurement capabilities, $I_{\mathrm{accessible}}$ approaches $S_{\mathrm{content}}$ and $S_{\mathrm{thermo}}$ approaches zero. For such an observer the second law trivializes—which matches the standard observation that an omniscient demon does not see thermodynamic irreversibility.

The second law is statistical, not deterministic. The expectation in (20) is essential. The result is a statement about typical behavior under typical Hamiltonians and typical initial states. Atypical configurations—states near a Poincaré recurrence, integrable Hamiltonians, conserved quantities preventing thermalization—can violate the inequality. The typical statement formulation matches the standard understanding of the second law in statistical mechanics and makes precise the sense in which the second law is a probabilistic statement.

### 3.5. Distinction with the Boltzmann Entropy

A natural question raised by the observer-relative character of $S_{\mathrm{thermo}}$ is its relation to the Boltzmann entropy $S_{B}=k_{B}\ln W$, which is not explicitly observer-relative.

The Boltzmann entropy is defined with respect to a chosen coarse-graining: *W* is the number of microstates compatible with the specified macrostate (typically defined by total energy, particle number, or extensive conserved quantities). The choice of coarse-graining is itself an implicit specification of what an observer can or chooses to distinguish. In that sense $S_{B}$ carries an observer-like component, but the observer is conventionally the thermodynamic experimenter with access to macroscopic variables, and the dependence on this choice is rarely made explicit.

In our taxonomy, $S_{B}$ aligns most naturally with $S_{\mathrm{content}}$ in the regime where the reduced state $\rho_{R}$ is approximately a microcanonical or canonical thermal state corresponding to the chosen macrostate. The number of microstates *W* corresponds, quantum-statistically, to the dimension of the typical subspace of states compatible with the macroscopic constraints. The relation $S_{B}=\ln W$ is then recovered as the von Neumann entropy of the maximally mixed state on that subspace.

In the idealized observer limit where $I_{\mathrm{accessible}}\to S_{\mathrm{content}}$, $S_{\mathrm{thermo}}$ goes to zero, whereas $S_{B}$ is not affected—because the macrostate-based coarse-graining is independent of any specific observer’s access. The two quantities answer different operational questions: $S_{B}$ counts microstates compatible with a coarse-graining; $S_{\mathrm{thermo}}$ measures the residual ignorance of a specific observer given the measurements performed. They coincide in the standard thermodynamic case where the observer’s measurement protocol matches the macrostate-based coarse graining and diverge in general operational settings, including the simple two-spin example.

### 3.6. Comparison with Existing Derivations

The derivation presented here is in the spirit of the information-theoretic approach to thermodynamics initiated by Jaynes [10]. It incorporates the typicality results of Popescu, Short, Winter [30] and Goldstein et al. [31], and follows the modern formulation of quantum thermodynamics summarized by Goold et al. [13]. What is new in the present formulation is the explicit operational identification of the thermodynamic entropy as $S_{\mathrm{content}}-I_{\mathrm{accessible}}$, and the resulting demonstration that the second law factors into two distinct ingredients, each with independent justification. This explicit factorization is what makes the conceptual structure of the derivation transparent in the taxonomy.

## 4. Applications

We illustrate the value of the taxonomy by examining two contexts in which the conflation of the three entropies has caused interpretive confusion: black hole thermodynamics and Jacobson’s derivation of Einstein’s equations.

### 4.1. Which Entropy Is the Bekenstein–Hawking Entropy?

The Bekenstein–Hawking entropy (1) is, in the framework of this paper, identified with $S_{\mathrm{capacity}}$ of the region enclosed by the horizon:

(21) $$S_{BH}\;=\;S_{\mathrm{capacity}}\left(R_{\mathrm{BH}}\right)\;=\;\frac{A}{4\ell_{P}^{2}}.$$

This identification is an interpretive choice, not a derivation, and we acknowledge that it is not the only available reading.

In approaches that derive black hole entropy from microstate counting—most notably string theory for supersymmetric black holes [34,35] and loop quantum gravity for spherically symmetric horizons [36,37,38]—$S_{BH}$ is interpreted as the logarithm of the dimension of a microscopic Hilbert space. This reading is more naturally a content-type entropy than a capacity-type bound. Likewise, in AdS/CFT and holography more broadly, the Ryu–Takayanagi prescription [25] and its quantum corrections through the quantum extremal surface formula [27,39] relate geometric areas to entanglement entropies in a highly non-trivial way, in many cases supporting the saturation $S_{\mathrm{content}}=S_{\mathrm{capacity}}$ at horizons. Recent calculations on the time evolution of entanglement entropy in gravitational collapse [40], on horizon entanglement in regular black hole configurations [41], and on entanglement entropy in quantum-corrected black hole metrics [42,43] provide explicit examples where geometric area laws emerge from quantum-informational quantities.

The present taxonomy is compatible with these viewpoints but adopts a different conceptual emphasis. Within our framework:

- $S_{BH}$ as $S_{\mathrm{capacity}}$: a geometric upper bound, derived from semiclassical gravity and assumed as a structural input.
- $S_{\mathrm{content}}\left(R_{\mathrm{BH}}\right)$: the actual fine-grained entanglement entropy, generally less than $S_{BH}$ for a black hole formed from a pure state, approaching $S_{BH}$ for an equilibrated old black hole and for situations described by the saturation regime of holographic prescriptions.
- The interpretive question of whether $S_{BH}$ is the von Neumann entropy of an underlying microstate—or whether $S_{BH}$ bounds that entropy—is precisely the saturation question.

This identification clarifies the information paradox [8]. The paradox can be reformulated as the question of whether $S_{\mathrm{content}}\left(R_{\mathrm{BH}}\right)=S_{\mathrm{capacity}}\left(R_{\mathrm{BH}}\right)$ holds throughout black hole evaporation, and whether the final state of the combined system (black hole remnant plus radiation) is pure or mixed. The taxonomy does not resolve this question, and it does not contest the microstate-counting interpretations where they are operationally available. What it provides is a framework in which the question can be posed without conflating the two distinct entropies involved.

The Hawking radiation process is naturally described in our taxonomy as a transfer of $S_{\mathrm{content}}$ from the black hole to the exterior, accompanied by a decrease in $S_{\mathrm{capacity}}$ as the horizon area shrinks. The area law dA/dt≥0 for classical processes is then a statement about $S_{\mathrm{capacity}}$, not about $S_{\mathrm{thermo}}$. The two coincide only under specific assumptions about the relation between content saturation and observer access. The generalized second law [24,44], which combines $S_{BH}$ with the entropy of matter outside the horizon, is consistent with the present framework provided the matter entropy is identified with $S_{\mathrm{content}}$ of the exterior region.

### 4.2. Which Entropy Enters the Clausius Relation in Jacobson’s Derivation?

In Jacobson’s derivation [9], the Einstein equations are obtained from the Clausius relation δQ=TdS applied to local Rindler horizons, with *S* identified with the area entropy and *T* with the Unruh temperature. We can now ask: which of the three entropies is *S*?

Within the present framework, *S* in Jacobson’s derivation is $S_{\mathrm{capacity}}$. The reasoning is direct: Jacobson uses $S=A/(4\ell_{P}^{2})$, which is by definition $S_{\mathrm{capacity}}$. The Clausius relation then expresses how the capacity of the local horizon changes in response to the energy flux through it.

This identification raises a legitimate question. The Clausius relation in standard thermodynamics is a relation among genuinely thermodynamic quantities: heat, temperature, and thermodynamic entropy. If *S* in Jacobson’s derivation is $S_{\mathrm{capacity}}$ rather than $S_{\mathrm{thermo}}$, the use of the Clausius relation needs justification.

The justification, in the present framework, lies in the local equilibrium structure of the Rindler observer. A Rindler observer at fixed proper acceleration sees the quantum vacuum as a thermal state at the Unruh temperature [45]. In this thermal regime, $S_{\mathrm{content}}\left(R\right)$ of the Rindler wedge is approximately equal to $S_{\mathrm{thermo}}\left(R,O_{\mathrm{Rindler}}\right)$ for the Rindler observer, and both are approximately related to $S_{\mathrm{capacity}}\left(R\right)$ at the horizon through the holographic saturation conjectured at horizons. The Clausius relation in Jacobson’s derivation can therefore be read as a relation among quantities that approximately coincide in the Rindler thermal regime, with $S_{\mathrm{capacity}}$ as the geometrically natural variable for the formulation.

The deeper question of why this approximate coincidence should hold, and what its breakdown would imply, belongs to the active research program on the thermodynamic interpretation of gravity [46,47,48]. The taxonomy proposed here does not resolve this question; it makes precise which entropies are involved and which coincidence is being invoked.

This reading is consistent with subsequent developments by Padmanabhan [46] and Verlinde [47], where gravitational dynamics emerges from informational or thermodynamic considerations on horizons. It does not modify Jacobson’s derivation; it disambiguates which of the three entropies is the relevant one.

### 4.3. Brief Comment on Decoherence

In standard treatments of decoherence [49,50], an open quantum system in interaction with an environment loses its coherence as entanglement with the environment grows. In our framework, this process reads as follows: $S_{\mathrm{content}}$ of the system increases as it becomes entangled with the environment, while $I_{\mathrm{accessible}}$ for an observer measuring only the system in a fixed basis remains bounded by the apparatus capability $I_{\max}$. The thermodynamic entropy $S_{\mathrm{thermo}}$ of the system therefore increases, in agreement with the standard interpretation of decoherence as an effective thermodynamic process.

The increase in $S_{\mathrm{thermo}}$ during decoherence depends on the choice of observer’s measurement basis. An observer measuring in the pointer basis selected by decoherence [51] sees minimal residual entropy increase, while an observer measuring in a basis orthogonal to the pointer basis sees maximal increase. This relativity matches the standard observation that decoherence does not by itself produce a unique classical outcome; it produces an apparent mixture relative to a specified basis. The taxonomy makes this dependence explicit. This is not a new result: it is a consistency check that the proposed taxonomy reproduces the expected behavior in a well-understood regime.

## 5. Relation to Existing Literature

We summarize here how the proposed taxonomy relates to existing approaches and identify what we believe goes beyond established terminology.

### 5.1. Information-Theoretic Approaches to Entropy

The view that thermodynamic entropy is an observer-relative information-theoretic quantity is associated with Jaynes [10]. It was developed in the analysis of Maxwell’s demon by Szilard [52], Landauer [11], and Bennett [12], and articulated in modern form in quantum thermodynamics [13,14,53]. The taxonomy proposed here belongs to this tradition. What we add is the explicit separation of $S_{\mathrm{thermo}}$ from $S_{\mathrm{content}}$ as a definitional relation, rather than as an implicit identification. This separation is what makes the role of the observer in the derivation of the second law operationally visible.

### 5.2. Subsystem and Entanglement Entropy

The identification of $S_{\mathrm{content}}$ as the fine-grained reduced-state entropy is standard in quantum information theory [23,54]. The role of entanglement entropy in semiclassical gravity is the subject of an extensive literature [25,26,27,39]. Our use of $S_{\mathrm{content}}$ as a concept separate from $S_{\mathrm{capacity}}$ aligns with the holographic literature, where the saturation between the two is a non-trivial dynamical statement rather than a definitional identity.

### 5.3. Geometric and Area Law Entropies

The Bekenstein–Hawking entropy [5,6], the Bekenstein bound [18], the holographic bound [19,20], the Bousso covariant bound [21,22], and the Wald entropy in higher-curvature gravity [55] are geometric quantities, related but not identical. In our taxonomy, $S_{\mathrm{capacity}}$ is meant to capture the conceptual content they share: a geometric upper bound on the information content of a region. The proper formulation of $S_{\mathrm{capacity}}$ in covariant settings is given by the established literature on covariant entropy bounds, which is not modified by our framework.

### 5.4. Recent Entanglement-Based Black Hole Calculations

Several recent works have computed entanglement entropy in specific gravitational settings: the time evolution during gravitational collapse [40], horizon entanglement in regular black hole metrics [41], entanglement in quantum-corrected black hole solutions [42], and entanglement structure in cosmological contexts [43]. These calculations operate on the content entropy side of our taxonomy: they compute $S_{\mathrm{content}}$ for specific physical configurations and compare it to the geometric area $S_{\mathrm{capacity}}$. The results give explicit examples where geometric area laws emerge from quantum-informational quantities, support the structural inequality (6), and clarify the conditions under which saturation occurs. Our framework is intended as a tool for organizing such results, not for replacing or modifying them.

### 5.5. What Is New in the Present Taxonomy

We can now identify what the taxonomy adds beyond known terminology:

1. The explicit decomposition $S_{\mathrm{thermo}}=S_{\mathrm{content}}-I_{\mathrm{accessible}}$ as a definitional relation, rather than as an implicit identification within specific operational protocols.
2. The factorization of the second law into two structurally independent ingredients (typical growth of $S_{\mathrm{content}}$ and boundedness of $I_{\mathrm{accessible}}$), each justified independently.
3. The taxonomic separation between $S_{\mathrm{capacity}}$ as a structural input and $S_{\mathrm{content}}$ as a state-dependent observable, with their relation expressed as a structural inequality rather than as a definitional identity.
4. The disambiguation of which entropy is involved in foundational results (Bekenstein–Hawking, Clausius relation in Jacobson’s derivation, decoherence) where the term “entropy” is sometimes used without precise specification.

None of these additions claims new physical content. They make explicit, at the level of definitions, distinctions that are present but often implicit in the literature.

## 6. Discussion and Limitations

The taxonomy proposed in this paper is intentionally minimal. We have not introduced new physical postulates beyond those already established in the literature. The contribution of the present work is to combine these ingredients into a single coherent operational framework and to draw out the consequences.

We highlight several limitations of the framework.

### 6.1. Definition of $I_{\mathrm{accessible}}$

The accessible information $I_{\mathrm{accessible}}$ is defined operationally as the mutual information that the observer can in principle extract through specified measurements. The two-spin example of Section 2.6 shows how this quantity is computed explicitly for a concrete observer. A more fundamental theory of observation would specify, for example, the maximal accessible information for an observer described by a finite-dimensional measurement apparatus interacting with the system through a specified quantum channel. The present paper does not develop this finer characterization. The structural inequality $I_{\mathrm{accessible}}\leq I{\left(R:O\right)}_{\rho }$ suffices for the emergent second law argument, but more refined applications—specific quantum measurement protocols, continuous quantum measurements, thermodynamic processes with finite-time constraints—would benefit from a sharper operational definition.

### 6.2. Covariance and Gauge Structure

The definitions of Section 2 are formulated in a fixed background geometry, with a fixed tensor product decomposition of the global Hilbert space. In gauge theories and gravity this decomposition is not generally valid: local subsystems carry edge modes [15], and the algebra of observables on a region is typically not of type I in the von Neumann algebraic classification [16]. The proper framework in those cases involves type II or type III algebras with corresponding modifications of entropy definitions [56,57]. Edge-mode contributions to entanglement entropy are physically meaningful and modify the relation between $S_{\mathrm{content}}$ and the area of ∂R. The taxonomy as presented here implicitly assumes a regime where these subtleties can be neglected: lattice regularization, finite-dimensional approximations, or specific gauge-fixing. Extension to fully covariant and algebraic settings is a natural direction for further work and is the subject of an active research program in quantum gravity [25,27,44].

### 6.3. Domain of Validity of the Typicality Result

The emergent second law (20) is a statement about typical behavior in the operational regime specified in Section 3.1. Systems outside that regime—integrable systems with conserved charges, many-body localized systems, systems with strong dynamical constraints—may behave atypically, and the second law as derived here may not apply or may apply only in modified form. The relevant timescales depend strongly on the Hamiltonian, the subsystem size, and the environment dimension. The Page time terminology is used here in its generic sense of the timescale at which entanglement saturates, with the understanding that the specific value depends on context.

### 6.4. Saturation of the Holographic Bound

The structural inequality $S_{\mathrm{content}}\leq S_{\mathrm{capacity}}$ is conjectured to hold generally in consistent theories of quantum gravity, with saturation at horizons in semiclassical treatments. Whether the saturation is exact, approximate, or holds only in specific dynamical regimes is an open question, with substantial recent literature [25,27,41,42]. The taxonomy does not depend on the specific resolution of this question; it provides a vocabulary in which the question is naturally posed.

### 6.5. Status as a Clarification

The taxonomy proposed here is conceptual. It does not introduce new dynamical equations, does not predict new physical phenomena, and does not modify any established result. Its value lies in providing a clearer vocabulary for foundational discussions where the conflation of the three entropies has caused confusion. It is intended as a useful clarification, not as a new theory.

## 7. Conclusions

We have proposed an operational distinction among three entropy-like quantities in physics: a geometric capacity entropy $S_{\mathrm{capacity}}$, a microscopic content entropy $S_{\mathrm{content}}$, and an observer-relative thermodynamic entropy $S_{\mathrm{thermo}}$. The three are linked by the structural inequality $S_{\mathrm{content}}\leq S_{\mathrm{capacity}}$ and by the operational definition $S_{\mathrm{thermo}}=S_{\mathrm{content}}-I_{\mathrm{accessible}}$. The operational meaning of $I_{\mathrm{accessible}}$ has been illustrated by an explicit two-spin example, in which the same physical state yields different thermodynamic entropies for observers with different measurement bases.

The principal consequence of keeping the three quantities distinct is that the second law of thermodynamics, formulated as a statement about $S_{\mathrm{thermo}}$, emerges as a typical consequence of two ingredients: the generic growth of $S_{\mathrm{content}}$ under unitary system-environment coupling, and the boundedness of observer access. Within the operational regime specified, the second law does not require an independent postulate. It is a consequence of quantum-informational and operational facts that have independent justification. The framework does not claim to derive the second law in its broadest empirical form; it shows that, in the regime stated, the reduction is complete.

The taxonomy further clarifies which entropy is the relevant one in two established contexts. The Bekenstein–Hawking entropy of black holes is identified, in our framework, with $S_{\mathrm{capacity}}$, while acknowledging that microstate-counting and holographic approaches naturally read $S_{BH}$ as a content-type entropy via the saturation of the holographic bound. The entropy entering the Clausius relation in Jacobson’s derivation of Einstein’s equations is also $S_{\mathrm{capacity}}$, with the Clausius relation justified through the approximate coincidence of $S_{\mathrm{content}}$, $S_{\mathrm{thermo}}$, and $S_{\mathrm{capacity}}$ in the Rindler thermal regime. These identifications do not modify the underlying results—they disambiguate their content.

The framework is conceptual. It does not constitute a new dynamical theory, and it does not replace any established result. Its value is in providing a more careful vocabulary for foundational discussions, particularly in informational approaches to physics, where the distinction among the three entropies is operationally significant. Whether this clarification proves useful in specific applications—in particular, in the resolution of long-standing ambiguities in black hole information dynamics and in emergent gravity—is a question for subsequent work.

## Figures and Tables

**Table 1:** The three entropies considered in this paper.

Quantity	Definition	Interpretation	Reference
$S_{\mathrm{capacity}}$	$A\left(R\right)/\left(4\ell_{P}^{2}\right)$	Geometric bound	[5,6,21]
$S_{\mathrm{content}}$	$-\mathrm{Tr}(\rho_{R}\ln\rho_{R})$	Fine-grained reduced-state entropy	[4]
$S_{\mathrm{thermo}}$	$S_{\mathrm{content}}-I_{\mathrm{accessible}}$	Observer-relative ignorance	[10,11]

## Data Availability

No new data were created or analyzed in this study. Data sharing is not applicable to this article.
